# *catena*-Poly[[diaqua­cobalt(II)]bis­[μ-2-(4-carboxyl­atophen­yl)-4,4,5,5-tetra­methyl-4,5-dihydro-1*H*-imidazol-1-oxyl 3-oxide]]

**DOI:** 10.1107/S1600536811009925

**Published:** 2011-03-26

**Authors:** Rong Rong, Ge Gao, Lili Zhu

**Affiliations:** a Department of Chemistry, Key Laboratory of Medicinal Chemistry for Natural Resources, Ministry of Education, Yunnan University, Kunming 650091, People’s Republic of China; b Ordered Matter Science Research Center, Department of Chemistry and Chemical Engineering, Southeast University, Nanjing 210096, People’s Republic of China; c School of Chemistry and Materials Science, Huaibei Normal University, Anhui 235000, People’s Republic of China

## Abstract

In the title compound, [Co(C_14_H_16_N_2_O_4_)_2_(H_2_O)_2_]_*n*_, the Co^II^ atom, lying on an inversion center, is coordinated by six O atoms in a distorted octa­hedral geometry. The Co^II^ atoms are bridged by the nitronyl nitroxide ligands into a tape-like structure along the *b* axis. The tapes are further connected by O—H⋯O hydrogen bonds, forming a layer parallel to the *bc* plane.

## Related literature

For related structures, see: Caneschi *et al.* (1993 ▶); Luneau *et al.* (1998 ▶). For the synthesis of [Co(C_5_H_9_O_2_)_2_(H_2_O)_2_], see: Mehrotra & Bohra (1983 ▶). For the synthesis of 2-(4-carb­oxy­phen­yl)-4,4,5,5-tetra­methyl-4,5-dihydro-1*H*-imidazol-1-oxyl-3-oxide, see: Schiødt *et al.* (1996 ▶). 
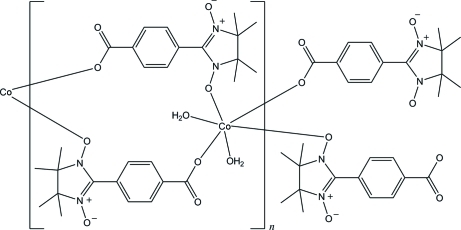


## Experimental

### 

#### Crystal data

[Co(C_14_H_16_N_2_O_4_)_2_(H_2_O)_2_]
*M* *_r_* = 647.54
Monoclinic, 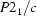

*a* = 13.548 (3) Å
*b* = 9.2054 (18) Å
*c* = 12.549 (3) Å
β = 115.17 (3)°
*V* = 1416.5 (5) Å^3^
*Z* = 2
Mo *K*α radiation
μ = 0.67 mm^−1^
*T* = 293 K
0.43 × 0.42 × 0.20 mm

#### Data collection

Rigaku SCXmini diffractometer
Absorption correction: multi-scan (*CrystalClear*; Rigaku, 2005 ▶) *T*_min_ = 0.240, *T*_max_ = 0.428
14419 measured reflections
3241 independent reflections
2050 reflections with *I* > 2σ(*I*)
*R*_int_ = 0.121

#### Refinement

*R*[*F* ^2^ > 2σ(*F* ^2^)] = 0.070
*wR*(*F* ^2^) = 0.169
*S* = 1.04
3241 reflections
196 parameters
H-atom parameters constrained
Δρ_max_ = 0.54 e Å^−3^
Δρ_min_ = −0.54 e Å^−3^

### 

Data collection: *CrystalClear* (Rigaku, 2005 ▶); cell refinement: *CrystalClear*; data reduction: *CrystalClear*; program(s) used to solve structure: *SHELXS97* (Sheldrick, 2008 ▶); program(s) used to refine structure: *SHELXL97* (Sheldrick, 2008 ▶); molecular graphics: *SHELXTL* (Sheldrick, 2008 ▶); software used to prepare material for publication: *SHELXTL*.

## Supplementary Material

Crystal structure: contains datablocks I, global. DOI: 10.1107/S1600536811009925/is2683sup1.cif

Structure factors: contains datablocks I. DOI: 10.1107/S1600536811009925/is2683Isup2.hkl

Additional supplementary materials:  crystallographic information; 3D view; checkCIF report

## Figures and Tables

**Table 1:** Hydrogen-bond geometry (Å, °)

*D*—H⋯*A*	*D*—H	H⋯*A*	*D*⋯*A*	*D*—H⋯*A*
O5—H1*O*5⋯O2^i^	0.87	2.31	2.736 (4)	111
O5—H2*O*5⋯O2^ii^	0.85	2.40	2.886 (4)	117

Symmetry codes: (i) 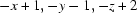; (ii) 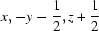.

**Fig. 1.**
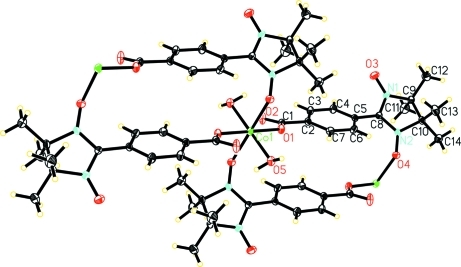
The molecular structure of the title compound, with the atom-numbering scheme and all hydrogen atoms. Displacement ellipsoids are drawn at the 30% probability level.

**Fig. 2.**
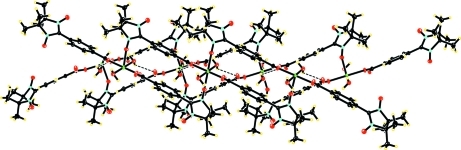
A packing view of the title compound. Hydrogen bonds are shown as dashed lines.

## supplementary crystallographic information

### Comment

The combination of paramagnetic metal ions and organic nitronyl nitroxides has attracted much more attention in the last decades, because of their intriguing structural, magnetic, and spectral properties (Caneschi *et al.*, 1993; Luneau *et al.*, 1998).

We herein report the crystal structure of a new complex revealed two ladder-like one-dimensional chains of repeating Co(NITpBA)_2_(H_2_O)_2_ units. As shown in Fig. 1, the central Co ion is located in a distorted octahedral environment. It is bonded to two oxygen atoms from carboxylic acid group, two oxygen atoms from nitroxide group and two oxygen atoms from two water atoms. Each radical coordinates the next Co(II) ion in the opposite fashion thus forming chains. There are intermolecular hydrogen bonds between the coordinated water molecule and the non-coordinated carboxylic O2 atom (Table 1 and Fig. 2).

### Experimental

All reagents and chemicals were purchased from commercial sources. The carboxylates, Co(Me_3_CCO_2_)_2_(H_2_O)_2_, were prepared as described previously (Mehrotra & Bohra, 1983). The benzoic acid substituted nitronyl nitroxide radical NITpBAH) was synthesized according to the procedure previously described (Schiødt *et al.*, 1996). Co(Me_3_CCO_2_)_2_(H_2_O)_2_ (0.0290 g, 0.1 mmol) was dissolved with prolonged stirring at room temperature in acetonitrile (15 mL). Then a 10 mL dichloromethane solution of NITpBAH(0.0608 g, 0.2 mmol) was added dropwise with stirring. Both solutions were mixed while stirring was continued for 1 h, dark blue crystals suitable for X-ray analysis were obtained by slow evaporation at room temperature over several days.

### Refinement

Positional parameters of all H atoms bound to C were calculated geometrically (C—H = 0.93 or 0.96 Å) and the H atoms were refined as riding, with *U*_iso_(H) = 1.2*U*_eq_(C) or 1.5*U*_eq_(methyl C). The H atoms of water molecile were located in a difference Fourier map and fixed at the positions with *U*_iso_(H) = 1.5*U*_eq_(O).

### Crystal data

**Table:**

[Co(C_14_H_16_N_2_O_4_)_2_(H_2_O)_2_]	*F*(000) = 678
*M**_r_* = 647.54	*D*_x_ = 1.518 Mg m^−^^3^
Monoclinic, *P*2_1_/*c*	Mo *K*α radiation, λ = 0.71073 Å
Hall symbol: -P 2ybc	Cell parameters from 14419 reflections
*a* = 13.548 (3) Å	θ = 3.3–27.5°
*b* = 9.2054 (18) Å	µ = 0.67 mm^−^^1^
*c* = 12.549 (3) Å	*T* = 293 K
β = 115.17 (3)°	Prism, dark blue
*V* = 1416.5 (5) Å^3^	0.43 × 0.42 × 0.20 mm
*Z* = 2	

### Data collection

**Table:**

Rigaku SCXmini diffractometer	3241 independent reflections
Radiation source: fine-focus sealed tube	2050 reflections with *I* > 2σ(*I*)
graphite	*R*_int_ = 0.121
Detector resolution: 8.192 pixels mm^-1^	θ_max_ = 27.5°, θ_min_ = 3.3°
ω scans	*h* = −17→17
Absorption correction: multi-scan (*CrystalClear*; Rigaku, 2005)	*k* = −11→11
*T*_min_ = 0.240, *T*_max_ = 0.428	*l* = −16→16
14419 measured reflections	

### Refinement

**Table:**

Refinement on *F*^2^	Primary atom site location: structure-invariant direct methods
Least-squares matrix: full	Secondary atom site location: difference Fourier map
*R*[*F*^2^ > 2σ(*F*^2^)] = 0.070	Hydrogen site location: inferred from neighbouring sites
*wR*(*F*^2^) = 0.169	H-atom parameters constrained
*S* = 1.04	*w* = 1/[σ^2^(*F*_o_^2^) + (0.0616*P*)^2^ + 1.8428*P*] where *P* = (*F*_o_^2^ + 2*F*_c_^2^)/3
3241 reflections	(Δ/σ)_max_ < 0.001
196 parameters	Δρ_max_ = 0.54 e Å^−^^3^
0 restraints	Δρ_min_ = −0.54 e Å^−^^3^

### Special details

**Table:**

Geometry. All e.s.d.'s (except the e.s.d. in the dihedral angle between two l.s. planes) are estimated using the full covariance matrix. The cell e.s.d.'s are taken into account individually in the estimation of e.s.d.'s in distances, angles and torsion angles; correlations between e.s.d.'s in cell parameters are only used when they are defined by crystal symmetry. An approximate (isotropic) treatment of cell e.s.d.'s is used for estimating e.s.d.'s involving l.s. planes.
Refinement. Refinement of *F*^2^ against ALL reflections. The weighted *R*-factor *wR* and goodness of fit *S* are based on *F*^2^, conventional *R*-factors *R* are based on *F*, with *F* set to zero for negative *F*^2^. The threshold expression of *F*^2^ > σ(*F*^2^) is used only for calculating *R*-factors(gt) *etc*. and is not relevant to the choice of reflections for refinement. *R*-factors based on *F*^2^ are statistically about twice as large as those based on *F*, and *R*- factors based on ALL data will be even larger.

### Fractional atomic coordinates and isotropic or equivalent isotropic displacement parameters (Å^2^)

**Table:**

	*x*	*y*	*z*	*U*_iso_*/*U*_eq_	
O1	0.4450 (2)	−0.2949 (3)	0.9518 (2)	0.0274 (7)	
O2	0.4582 (3)	−0.2604 (3)	0.7820 (3)	0.0364 (8)	
O3	0.0826 (3)	0.3058 (4)	0.6289 (3)	0.0442 (9)	
O4	0.3405 (2)	0.4447 (3)	0.9959 (2)	0.0279 (7)	
O5	0.5531 (2)	−0.4483 (3)	1.1813 (2)	0.0297 (7)	
H1O5	0.6060	−0.5105	1.2077	0.045*	
H2O5	0.5186	−0.4578	1.2237	0.045*	
N1	0.1342 (3)	0.3787 (4)	0.7227 (3)	0.0267 (8)	
N2	0.2516 (3)	0.4405 (4)	0.8999 (3)	0.0233 (8)	
C1	0.4323 (3)	−0.2209 (4)	0.8615 (4)	0.0246 (9)	
C2	0.3798 (3)	−0.0740 (4)	0.8520 (4)	0.0234 (9)	
C3	0.3428 (3)	0.0019 (4)	0.7470 (4)	0.0264 (9)	
H3A	0.3513	−0.0380	0.6834	0.032*	
C4	0.2933 (4)	0.1363 (4)	0.7352 (4)	0.0284 (10)	
H4A	0.2687	0.1861	0.6640	0.034*	
C5	0.2805 (3)	0.1961 (4)	0.8299 (4)	0.0229 (9)	
C6	0.3184 (4)	0.1222 (4)	0.9369 (4)	0.0278 (10)	
H6A	0.3115	0.1634	1.0011	0.033*	
C7	0.3663 (3)	−0.0122 (4)	0.9470 (4)	0.0265 (9)	
H7A	0.3900	−0.0626	1.0177	0.032*	
C8	0.2253 (3)	0.3363 (4)	0.8166 (3)	0.0232 (9)	
C9	0.1031 (4)	0.5322 (4)	0.7365 (4)	0.0271 (10)	
C10	0.1582 (3)	0.5441 (4)	0.8726 (4)	0.0266 (10)	
C11	0.1545 (4)	0.6293 (5)	0.6754 (4)	0.0451 (13)	
H11A	0.1167	0.6166	0.5917	0.068*	
H11B	0.2298	0.6035	0.7010	0.068*	
H11C	0.1494	0.7290	0.6949	0.068*	
C12	−0.0192 (4)	0.5491 (6)	0.6821 (4)	0.0458 (13)	
H12A	−0.0472	0.5404	0.5981	0.069*	
H12B	−0.0377	0.6428	0.7018	0.069*	
H12C	−0.0506	0.4748	0.7118	0.069*	
C13	0.0892 (4)	0.4805 (5)	0.9312 (5)	0.0414 (12)	
H13A	0.1274	0.4910	1.0151	0.062*	
H13B	0.0760	0.3794	0.9117	0.062*	
H13C	0.0209	0.5312	0.9037	0.062*	
C14	0.1972 (4)	0.6944 (5)	0.9213 (4)	0.0392 (12)	
H14A	0.2299	0.6910	1.0058	0.059*	
H14C	0.1363	0.7600	0.8944	0.059*	
H14D	0.2500	0.7276	0.8946	0.059*	
Co1	0.5000	−0.5000	1.0000	0.0250 (3)	

### Atomic displacement parameters (Å^2^)

**Table:**

	*U*^11^	*U*^22^	*U*^33^	*U*^12^	*U*^13^	*U*^23^
O1	0.0352 (17)	0.0148 (15)	0.0341 (16)	0.0060 (12)	0.0165 (14)	0.0051 (12)
O2	0.058 (2)	0.0235 (17)	0.0439 (19)	0.0125 (15)	0.0372 (17)	0.0067 (14)
O3	0.041 (2)	0.035 (2)	0.0384 (19)	0.0023 (16)	−0.0002 (16)	−0.0109 (15)
O4	0.0256 (17)	0.0295 (16)	0.0250 (16)	0.0007 (13)	0.0074 (13)	−0.0007 (12)
O5	0.0334 (18)	0.0258 (16)	0.0338 (17)	0.0024 (13)	0.0181 (14)	0.0013 (13)
N1	0.028 (2)	0.0211 (19)	0.0277 (19)	0.0002 (15)	0.0080 (16)	−0.0034 (15)
N2	0.0203 (19)	0.0190 (18)	0.0300 (19)	0.0020 (14)	0.0101 (16)	−0.0012 (15)
C1	0.027 (2)	0.014 (2)	0.033 (2)	0.0016 (17)	0.013 (2)	0.0032 (17)
C2	0.024 (2)	0.015 (2)	0.032 (2)	0.0016 (17)	0.0124 (18)	0.0012 (17)
C3	0.034 (2)	0.020 (2)	0.030 (2)	0.002 (2)	0.0190 (19)	0.0013 (19)
C4	0.038 (3)	0.018 (2)	0.030 (2)	0.0046 (19)	0.016 (2)	0.0054 (18)
C5	0.021 (2)	0.013 (2)	0.033 (2)	0.0029 (16)	0.0094 (18)	0.0015 (17)
C6	0.035 (3)	0.020 (2)	0.029 (2)	−0.0006 (18)	0.014 (2)	−0.0041 (17)
C7	0.030 (2)	0.021 (2)	0.026 (2)	0.0008 (19)	0.0094 (18)	0.0049 (18)
C8	0.023 (2)	0.018 (2)	0.027 (2)	−0.0004 (17)	0.0092 (18)	−0.0028 (17)
C9	0.028 (2)	0.015 (2)	0.034 (2)	0.0058 (16)	0.009 (2)	0.0009 (17)
C10	0.027 (2)	0.018 (2)	0.035 (2)	0.0061 (17)	0.014 (2)	0.0010 (17)
C11	0.060 (4)	0.032 (3)	0.044 (3)	0.005 (2)	0.023 (3)	0.011 (2)
C12	0.035 (3)	0.035 (3)	0.053 (3)	0.011 (2)	0.004 (2)	−0.002 (2)
C13	0.041 (3)	0.042 (3)	0.052 (3)	0.005 (2)	0.030 (3)	0.006 (2)
C14	0.038 (3)	0.029 (3)	0.045 (3)	0.001 (2)	0.012 (2)	−0.008 (2)
Co1	0.0304 (5)	0.0162 (4)	0.0263 (4)	0.0034 (4)	0.0101 (4)	0.0022 (3)

### Geometric parameters (Å, °)

**Table:**

O1—C1	1.269 (5)	C6—H6A	0.9300
O1—Co1	2.026 (3)	C7—H7A	0.9300
O2—C1	1.244 (5)	C9—C12	1.508 (6)
O3—N1	1.274 (4)	C9—C11	1.525 (6)
O4—N2	1.292 (4)	C9—C10	1.550 (6)
O4—Co1^i^	2.199 (3)	C10—C14	1.514 (6)
O5—Co1	2.127 (3)	C10—C13	1.531 (6)
O5—H1O5	0.8657	C11—H11A	0.9600
O5—H2O5	0.8506	C11—H11B	0.9600
N1—C8	1.352 (5)	C11—H11C	0.9600
N1—C9	1.505 (5)	C12—H12A	0.9600
N2—C8	1.350 (5)	C12—H12B	0.9600
N2—C10	1.503 (5)	C12—H12C	0.9600
C1—C2	1.508 (5)	C13—H13A	0.9600
C2—C3	1.383 (5)	C13—H13B	0.9600
C2—C7	1.401 (6)	C13—H13C	0.9600
C3—C4	1.385 (6)	C14—H14A	0.9600
C3—H3A	0.9300	C14—H14C	0.9600
C4—C5	1.386 (6)	C14—H14D	0.9600
C4—H4A	0.9300	Co1—O1^ii^	2.026 (3)
C5—C6	1.394 (5)	Co1—O5^ii^	2.127 (3)
C5—C8	1.465 (5)	Co1—O4^iii^	2.199 (3)
C6—C7	1.378 (6)	Co1—O4^iv^	2.199 (3)
			
C1—O1—Co1	131.3 (3)	C14—C10—C13	109.6 (4)
N2—O4—Co1^i^	123.0 (2)	N2—C10—C9	99.8 (3)
Co1—O5—H1O5	96.3	C14—C10—C9	115.5 (4)
Co1—O5—H2O5	128.4	C13—C10—C9	113.3 (4)
H1O5—O5—H2O5	106.2	C9—C11—H11A	109.5
O3—N1—C8	126.3 (3)	C9—C11—H11B	109.5
O3—N1—C9	122.0 (3)	H11A—C11—H11B	109.5
C8—N1—C9	111.5 (3)	C9—C11—H11C	109.5
O4—N2—C8	125.3 (3)	H11A—C11—H11C	109.5
O4—N2—C10	123.6 (3)	H11B—C11—H11C	109.5
C8—N2—C10	110.8 (3)	C9—C12—H12A	109.5
O2—C1—O1	125.5 (4)	C9—C12—H12B	109.5
O2—C1—C2	118.9 (4)	H12A—C12—H12B	109.5
O1—C1—C2	115.6 (4)	C9—C12—H12C	109.5
C3—C2—C7	118.7 (4)	H12A—C12—H12C	109.5
C3—C2—C1	119.7 (4)	H12B—C12—H12C	109.5
C7—C2—C1	121.6 (4)	C10—C13—H13A	109.5
C2—C3—C4	121.1 (4)	C10—C13—H13B	109.5
C2—C3—H3A	119.5	H13A—C13—H13B	109.5
C4—C3—H3A	119.5	C10—C13—H13C	109.5
C3—C4—C5	119.6 (4)	H13A—C13—H13C	109.5
C3—C4—H4A	120.2	H13B—C13—H13C	109.5
C5—C4—H4A	120.2	C10—C14—H14A	109.5
C4—C5—C6	120.2 (4)	C10—C14—H14C	109.5
C4—C5—C8	119.8 (4)	H14A—C14—H14C	109.5
C6—C5—C8	120.0 (4)	C10—C14—H14D	109.5
C7—C6—C5	119.5 (4)	H14A—C14—H14D	109.5
C7—C6—H6A	120.2	H14C—C14—H14D	109.5
C5—C6—H6A	120.2	O1^ii^—Co1—O1	180.000 (1)
C6—C7—C2	120.9 (4)	O1^ii^—Co1—O5^ii^	91.43 (11)
C6—C7—H7A	119.5	O1—Co1—O5^ii^	88.57 (11)
C2—C7—H7A	119.5	O1^ii^—Co1—O5	88.57 (11)
N2—C8—N1	108.2 (3)	O1—Co1—O5	91.43 (11)
N2—C8—C5	125.7 (4)	O5^ii^—Co1—O5	180.000 (1)
N1—C8—C5	125.9 (4)	O1^ii^—Co1—O4^iii^	91.38 (11)
N1—C9—C12	110.6 (4)	O1—Co1—O4^iii^	88.62 (11)
N1—C9—C11	106.4 (4)	O5^ii^—Co1—O4^iii^	92.34 (11)
C12—C9—C11	111.1 (4)	O5—Co1—O4^iii^	87.66 (11)
N1—C9—C10	99.6 (3)	O1^ii^—Co1—O4^iv^	88.62 (11)
C12—C9—C10	114.4 (4)	O1—Co1—O4^iv^	91.38 (11)
C11—C9—C10	113.8 (4)	O5^ii^—Co1—O4^iv^	87.66 (11)
N2—C10—C14	111.9 (4)	O5—Co1—O4^iv^	92.34 (11)
N2—C10—C13	106.0 (3)	O4^iii^—Co1—O4^iv^	180.000 (1)

Symmetry codes: (i) *x*, *y*+1, *z*; (ii) −*x*+1, −*y*−1, −*z*+2; (iii) *x*, *y*−1, *z*; (iv) −*x*+1, −*y*, −*z*+2.

### Hydrogen-bond geometry (Å, °)

**Table:**

*D*—H···*A*	*D*—H	H···*A*	*D*···*A*	*D*—H···*A*
O5—H1O5···O2^ii^	0.87	2.31	2.736 (4)	111
O5—H2O5···O2^v^	0.85	2.40	2.886 (4)	117

Symmetry codes: (ii) −*x*+1, −*y*−1, −*z*+2; (v) *x*, −*y*−1/2, *z*+1/2.
